# Glial Promoter Selectivity following AAV-Delivery to the Immature Brain

**DOI:** 10.1371/journal.pone.0065646

**Published:** 2013-06-14

**Authors:** Georg von Jonquieres, Nadine Mersmann, Claudia Bettina Klugmann, Anne Editha Harasta, Beat Lutz, Orla Teahan, Gary David Housley, Dominik Fröhlich, Eva-Maria Krämer-Albers, Matthias Klugmann

**Affiliations:** 1 Translational Neuroscience Facility, Department of Physiology, School of Medical Sciences, University of New South Wales, New South Wales, Sydney, Australia; 2 Institute of Physiological Chemistry, University Medical Centre of the Johannes Gutenberg University, Mainz, Germany; 3 Department of Molecular Cell Biology, University of Mainz, Mainz, Germany; University of Kansas Medical Center, United States of America

## Abstract

Recombinant adeno-associated virus (AAV) vectors are versatile tools for gene transfer to the central nervous system (CNS) and proof-of-concept studies in adult rodents have shown that the use of cell type-specific promoters is sufficient to target AAV-mediated transgene expression to glia. However, neurological disorders caused by glial pathology usually have an early onset. Therefore, modelling and treatment of these conditions require expanding the concept of targeted glial transgene expression by promoter selectivity for gene delivery to the immature CNS. Here, we have investigated the AAV-mediated green fluorescent protein (GFP) expression driven by the myelin basic protein (MBP) or glial fibrillary acidic protein (GFAP) promoters in the developing mouse brain. Generally, the extent of transgene expression after infusion at immature stages was widespread and higher than in adults. The GFAP promoter-driven GFP expression was found to be highly specific for astrocytes following vector infusion to the brain of neonates and adults. In contrast, the selectivity of the MBP promoter for oligodendrocytes was poor following neonatal AAV delivery, but excellent after vector injection at postnatal day 10. To extend these findings obtained in naïve mice to a disease model, we performed P10 infusions of AAV-MBP-GFP in aspartoacylase (ASPA)-deficient mouse mutants presenting with early onset oligodendrocyte pathology. Spread of GFP expression and selectivity for oligodendrocytes in ASPA-mutants was comparable with our observations in normal animals. Our data suggest that direct AAV infusion to the developing postnatal brain, utilising cellular promoters, results in targeted and long-term transgene expression in glia. This approach will be relevant for disease modelling and gene therapy for the treatment of glial pathology.

## Introduction

Adeno-associated virus (AAV) vectors are the delivery platform of choice for central nervous system (CNS) gene transfer. The host selection of AAV vectors is determined by interactions between viral capsid proteins and specific receptors and co-receptors at the surface of target cells [1]. Therefore, AAV tropism is determined by the vector serotype [2] but also by vector purity [3], and the maturity of the host CNS [4]. While systemic AAV delivery results in transduction of both neurons and astrocytes [4], direct vector infusion to the CNS gene confers transgene expression primarily in neurons when ubiquitous promoters are employed [3], [5]–[7]. This has lead to the view that AAV vectors inherently transduce neurons with high preference when administered directly. However, this view has been challenged by proof-of-principle work suggesting that promoter selection massively influences the pattern of AAV-mediated transgene expression [7], [8]. In these studies, after AAV delivery to the adult rodent brain, the mouse myelin basic protein (MBP) and the glial fibrillary acidic protein (GFAP) promoters showed the respective oligodendroglial and astrocytic selectivity. It is not clear, however, if this approach can be adopted for somatic gene transfer to glia in the developing brain. The latter will be necessary to model or treat early onset diseases caused by a primary glial pathology.

Here, we investigated the expression patterns of the green fluorescent protein (GFP) reporter following intrastriatal delivery of AAV-MBP-GFP or AAV-GFAP-GFP to mice. We hypothesised that the numbers of permissive glia present at the time point of injection would improve the degree of promoter tropism. To address that, we aimed at identifying the promoter specificity following vector administration at postnatal day P0 (neonates), P10, and P90 (adults). Our data suggest that robust targeting of glia in the immature brain can be achieved by direct AAV injection. These findings will be important for disease modelling and gene therapy, or whenever efficient and selective transgene expression in glia is required.

## Materials and Methods

### Animals

#### Ethics statement

Experiments were approved by the local animal care committees (Landesuntersuchungsamt Koblenz, permit number 23177/G10-1-036; UNSW AEC 11/21B). All animals were single-housed in a temperature-controlled room (21–22°C; 49–55% humidity) with 12 h-light-dark-cycle (lights on 7:00–19:00), where food and water were available *ad libitum*. All *in vivo* experiments were performed in C57BL/6J mice.

#### Plasmid constructs

A rAAV plasmid backbone containing the woodchuck hepatitis virus post-transcriptional regulatory element (WPRE) and the bovine growth hormone polyadenylation sequence (bGHpA) flanked by AAV2 inverted terminal repeats was used to drive the cDNA encoding enhanced green fluorescent protein (GFP) under the control of the 1.1 kb CMV enhancer/chicken β-actin hybrid (CBA) promoter (pAAV-CBA-GFP). This backbone was digested with Acc65I-blunt/EcoRV to replace the CBA promoter with the 1.94 kb promoter of the mouse *myelin basic protein (mbp)* gene excised with NotI/BamHI (blunt) from pMBP-DTR [9] to create pAAV-MBP-GFP. pAAV-GFAP-GFP carrying the 2.2 kb human GFAP promoter [7] was kindly provided by Alexander Muravlev.

#### AAV vector production

The production of chimeric AAV1/2 vectors, carrying VP1, VP2 and VP3 capsid proteins from AAV1 and AAV2 at equal ratios, was performed as described previously [10], [11]. Briefly, human embryonic kidney 293 (HEK) cells were co-transfected with the AAV plasmid, the serotype-specific AAV helper plasmids, pH 21 and pRV-1, encoding *rep* and *cap* genes of AAV1 and AAV2, respectively, and the adenovirus helper plasmid (pFΔ6) by standard CaPO_4_ transfection. Cells were harvested 60 hours after transfection and vectors were purified using HiTrap heparin affinity columns (Sigma, St Louis, MO) and concentrated 3× by refilling with phosphate-buffered saline containing 1 mM MgCl_2_ and 2.5 mM KCl (PBS-MK) using Microsep™ Advanced Centrifugal Device 100K MWCO concentrators (Pall, Surry Hills, Australia). When the volume was at 250 µl the virus solution was collected, the concentrator rinsed with an equal volume of PBS-MK, and solutions were pooled and sterile filtered. Genomic titers were determined using the ABI StepOnePlus Real-Time PCR system (Applied Biosystems, Foster City, CA) with primers designed to WPRE [12].

#### Oligodendrocyte cultures

Primary cultures enriched in oligodendrocytes were prepared from embryonic day 14–16 mice as described [13], [14] with some minor modifications. Neural precursor cells growing on top of astrocyte monolayers were shaken off at day 13 after preparation (instead of day 15) and plated in modified Sato medium supplemented with B27, 1% horse serum, 10 ng/ml human recombinant platelet-derived growth factor (PDGF-AA), and 5 ng/ml basic fibroblast growth factor (bFGF) on Poly-L-Lysine coated coverslips (1×10^5^ cells/11 mm coverslip). The cells were allowed to differentiate for 4 days *in vitro* before virus infection with 1×10^9^ viral genomes (vg) and then kept for additional 8 days. The resulting cultures were enriched in oligodendrocytes (60–70% of cells) but contained detectable numbers of astrocytes (20–30%) and some neurons (10–15%). Transfection of oligodendrocyte-enriched cultures was performed immediately after shake-off from astrocyte monolayers utilizing AMAXA Biosystems technology according to the manufacturer's instructions (Amaxa®Nucleofector Kit, primary neurons; program O-005). Transfected cells (4×10^6^) were plated in a 6 cm dish containing poly-L-lysin-coated coverslips and analyzed after four days in culture by immunofluorescence (see below). The human oligodendroglial cell line MO3.13 [15] was grown in DMEM supplemented with 10% FCS, Pen/Strep. Cells were seeded at a density of 5×10^4^ per 11 mm glass coverslip before AAV infection with 1×10^9^ vg and then kept for additional eight days before fixation followed by EGFP-immunocytochemistry (see below).

#### AAV vector delivery in vivo

Adult or P10 mice were anaesthetised with either isoflurane (4% induction, then 1% maintenance with O_2_), or i.p. injections of a anaesthetic cocktail containing ketamine (40 mg/kg), xylazine (8 mg/kg) and acepromazine (0.5 mg/kg). Animals were then placed into a stereotaxic frame (Kopf instruments, Tujunga, CA). 1 µl of AAV-GFP vector, adjusted to 2×10^12^ vg/ml, was injected into the striatum (adults: +1.1 mm AP, −1.7 mm ML, −3.5 mm DV from bregma; P10: +4.0 mm AP, 1.7 mm ML, −2.3 mm DV from lambda). Vector delivery was performed at a rate of 150 nl/min using a microprocessor-controlled mini-pump (World Precision Instruments, Sarasota, FA, USA) with 34G beveled needles (World Precision Instruments) and the needle was left in place for five minutes prior to slowly retracting the needle from the brain.

For neonatal vector delivery (P0), pups (8–24 hrs after birth) were cryo-anesthetized and AAV administered as described [16]. Briefly, pups were immobilized by wrapping in a paper towel covered with wet-ice for 3–5 min and then positioned in a custom-made styrofoam mould for vector delivery into the striatum (+2.0 mm AP, −1.5 mm ML, −2.0 mm DV from lambda) using a hand-held 34G beveled needle (World Precision Instruments). The AAV infusion was micro-processor controlled (100 nl/s) and the needle was left in place for additional 10 s at the end of the injection to prevent backflow of virus containing solution. After the needle was retracted the pups were re-warmed on a heating matt and rolled in the bedding of their pre-warmed home cage before being returned to the dam.

#### Immunofluorescence detection of antigens in histological sections and dissociated cultured cells

Three weeks after vector infusion when AAV-mediated transgenic protein expression has peaked to remain at stable levels [10] mice were deeply anesthetized with pentobarbital and trans-cardially perfused with phosphate buffered saline (PBS), followed by 10% buffered neutral formalin (Sigma). Brains were post-fixed in PFA (2 h) and cryo-protected in 30% sucrose/PBS, then cut into 40 µm free-floating sections using a Leica CM 1850 cryostat (Leica Microsystems, Wetzlar, Germany), and stored at 4°C in cryoprotection solution (25% glycerin, 25% ethylene glycol and 50% PBS) until use. Cells were fixed in 10% BNF before being processed for immunocytochemistry. Fixed sections or cell cultures were washed with PBS, permeabilized with 0.1% TritonX-100 in PBS (PBS-Tx) and blocked in 4% normal horse serum (NHS) in PBS-Tx. Sections or cells were incubated overnight at 4°C with a combination of the following antibodies in 4% NHS in PBS-Tx: rabbit anti-aspa serum (1∶400, [17]; mouse anti-GFAP (1∶500, Sigma-Aldrich, MO); mouse anti-ALDH1L1 (1∶500; NeuroMab, CA); mouse anti-NeuN (1∶500; Millipore, MA); rabbit anti-EGFP serum (1∶500, made in-house); mouse anti-EGFP (1∶500, Roche, Switzerland), mouse anti-O4 and rat anti-L1 (both undiluted, kind gifts of J. Trotter).

Sections/cells were washed with PBS and incubated with appropriate Alexa-488/594 conjugated secondary antibodies (1∶1000, Invitrogen, CA) for 2 h at room temperature in 4% NHS in PBS-Tx. After three washes in PBS-Tx specimen were mounted on slides and coverslipped with Mowiol (Calbiochem, Germany). Fluorescence was visualized using a Leica DMRA inverted microscope (Leica Microsystems, Wetzlar, Germany) or a Zeiss Z1 AxioExaminer NLO710 confocal microscope (Carl Zeiss MicroImaging, Germany).

#### Quantification of cell-type specific transgene expression and viral spread

Generally, the spread of AAV-mediated transgene expression will be referred to as vector spread. Three weeks following vector delivery to neonates (P0), P10 or adult (P90) mice (n = 3 per time point) the animals were sacrificed. Quantitative analyses were adapted from Markakis et al. [18]. The identity of GFP-expressing cells in the striatum was examined by double-immunofluorescence with antibodies against GFP and ASPA (oligodendrocytes), NeuN (neurons), or ALDH1L1 (astrocytes) in confocal images at 20× magnification. The percentage of GFP-expressing cells per cell-type was determined by counting at least 50 cells from three non-adjacent sections for a total of at least 150 GFP^+^ cells (n = 3). Relative quantification of the GFP expressing cell-types was performed using the ‘cell counter’ plugin for ImageJ version 1.45 k (NIH). In addition, the percentage of all cells of a population expressing GFP was calculated. To measure the vector spread images of every 8^th^ section expressing GFP were taken at 2.5× magnification. The total area of GFP expression was determined using the measurement tool integrated in the Zen 2010 imaging software (Carl Zeiss MicroImaging, Germany) and plotted in GraphPad Prism 5 software (La Jolla, CA). The volume of the vector spread was estimated by multiplying the area of GFP-immunoreactivity by the section thickness and the sampling interval. For quantitation of the vector spread relative to the whole brain volume we normalized the absolute vector spread to the brain volume (mm^3^) at the respective age for C57BL6/J mice adopted from Zhang et al. [19].

#### Statistics

All graphs and statistical analyses were done with GraphPad Prism 5 software (La Jolla, CA). Quantitative measures were analysed by ANOVA followed by Tukey's HSD test. Volume of vector spread was analysed by 2-way ANOVA and Bonferroni post-test). Values are presented as the mean±s.e.m.

## Results

### Cellular promoters target AAV1/2-mediated transgene expression to selected neural populations *in vitro*

**Figure 1:**
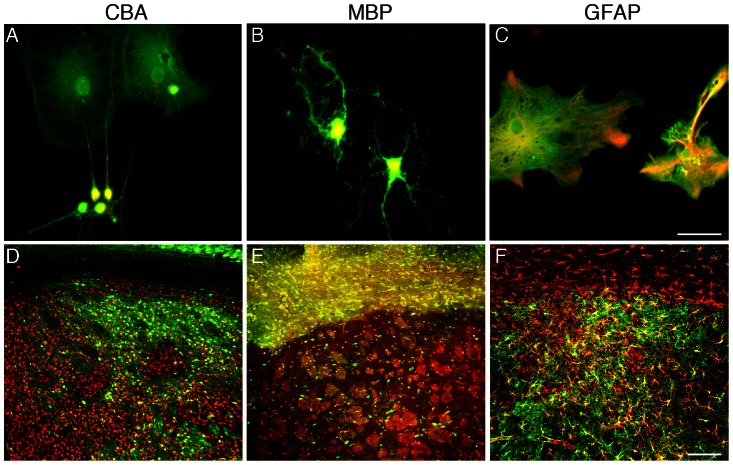
Promoter selectivity targets transgene expression to specific neural cell types *in vitro* and in the adult brain. AAV vectors (1×10^9^ vg) were used to express GFP driven by the indicated promoters in enriched primary oligodendrocyte cultures (A–C). For in vivo studies vectors (2×10^9^ vg) were injected in the striatum of adult mice (D–F). Representative images of results by double-immunocytochemistry for GFP (green) and cell-type specific markers (red) illustrate promoter selectivity. A, In primary cultures AAV-CBA-GFP expressed in all NeuN^+^ neurons. In addition, NeuN-negative astrocytes (top in picture) showed GFP immunoreactivity. B, AAV-MBP-GFP-mediated GFP-expression was restricted to ASPA^+^ oligodendrocytes in vitro. C, AAV-GFAP-GFP transduction resulted in GFP immunoreactivity limited to cultured GFAP^+^ astrocytes. D, CBA promoter-controlled GFP expression was highly specific to neurons in vivo. E, The MBP promoter was selective for forebrain oligodendrocytes. F, The GFAP promoter drove GFP specifically in astrocytes. Representative results from three independent experiments are shown. Bars: A–C, 50 µm; D–F, 100 µm.

For our initial experiments on targeted transgene expression by AAV, we investigated the properties of the MBP and GFAP promoters to control expression of the GFP reporter in oligodendrocyte-enriched primary cultures containing some astrocytes and neurons. The CBA promoter was included as a control because of its known neuron-specificity after direct AAV injections to the adult CNS [5], [7]. The corresponding AAV constructs were packaged into chimeric AAV1/2. This serotype was selected because it has shown widespread striatal transduction in previous studies [5], [20]. Double immunofluorescence detection of the reporter and cell type specific markers (ASPA for oligodendrocytes; GFAP for astrocytes; NeuN for neurons) was performed to assess cell type-specific transgene expression (Fig. 1A–C). AAV-CBA-GFP mediated transgene expression was observed in neurons and astrocytes but not oligodendrocytes (Fig. 1A). The MBP-promoter restricted GFP expression to oligodendrocytes (Fig. 1B), and the GFAP promoter was selective for astrocytes (Fig. 1C). We obtained similar results results using AAV1 or AAV8 (not shown). These data suggested that transduction might occur in all CNS cell types but cell type-specificity of transgene expression is conferred by promoter selectivity, rather than capsid tropism. To address this, we transfected the naked AAV-plasmid DNA constructs (pAAV-MBP-GFP and pAAV-CBA-GFP) in enriched oligodendrocyte cultures and performed immunocytochemistry for cell type-specific markers and the GFP reporter. Transfection of pAAV-CBA-GFP resulted in transgene expression in neurons (Figure S1) but not in oligodendrocytes (not shown). In contrast, MBP-driven GFP expression was restricted to oligodendrocytes. Since plasmid transfection warrants uptake of DNA by all cells types, these result suggests that the MBP promoter is both essential and sufficient for AAV-mediated transgene expression in oligodendrocytes.

### Widespread and selective transgene expression following AAV1/2-delivery to the adult brain

**Figure 2:**
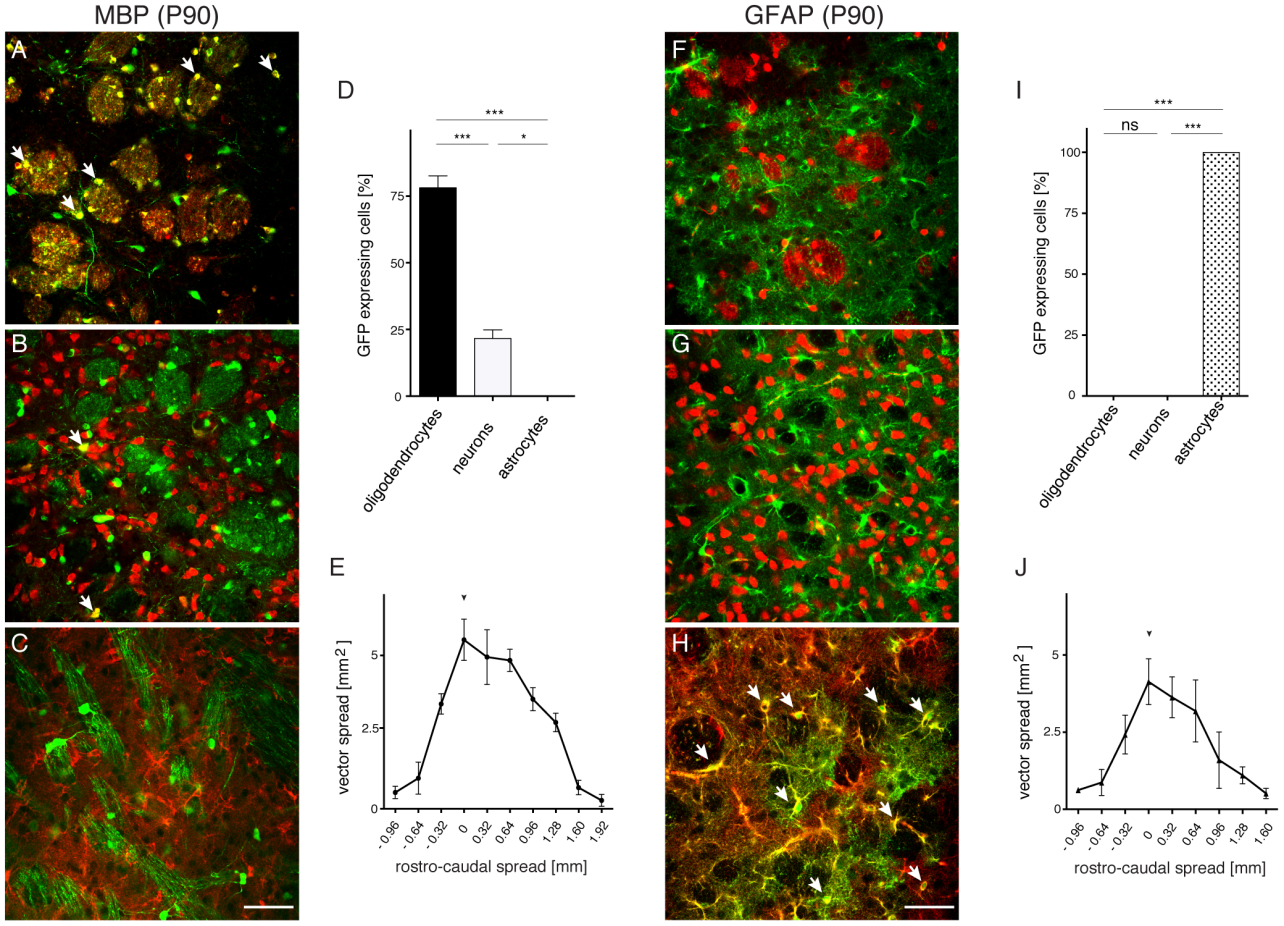
Quantification of glial promoter selectivity and vector spread after AAV delivery to adult mice. Three weeks prior to analysis 2×10^9^ vg AAV-MBP-GFP (A–E) or AAV-GFAP-GFP (F–J) were injected into the dorsal striatum of adult mice (n = 3). Higher magnification views of the striatum following detection of GFP with either ASPA (A,F), NeuN (B,G) or ALDH1L1 (C,H) was performed to identify the GFP^+^ cell-types. Quantification of results after AAV-MBP-GFP injection showed the vast majority of transgene-expressing cells were ASPA^+^ oligodendrocytes, followed by a smaller fraction representing NeuN^+^ neurons (D). In contrast, AAV-GFAP-GFP-mediated transgene expression was strictly astrocytic (I). The vector spread, determined by monitoring transgene expression in the rostro-caudal extension, was comparable for both vectors (E,J). Arrows in A,B,H indicate co-labelling. Bars: 50 µm.

Next, we injected the AAV1/2-GFP vectors into the striatum of adult mice and, three weeks later, performed double-stainings to detect GFP-immunoreactivity in neurons, oligodendrocytes, and astrocytes. Low power histological analysis (Fig. 1D–F) revealed widespread subcortical GFP-expression in grey matter (CBA), white matter tracts (MBP) and grey matter astrocytes (GFAP). Adult (P90) AAV-MBP-GFP and AAV-GFAP-GFP brains were then subjected to quantitative analyses of GFP-expression (Fig. 2). Markers for all subsequent experiments were as described above with the exception that we used the pan-astrocytic marker ALDH1L1 for reliable detection of grey matter astrocytes [21]. High power light microscopy shows MBP-controlled GFP-expression in striatal white and grey matter oligodendrocytes (Fig. 2A), and in some neurons (Fig. 2B). GFP immunoreactivity was not observed in astrocytes (Fig. 2C). Quantification of these results (Fig. 2D) showed that 78.2±4.4% of all GFP^+^ cells were oligodendrocytes, and 21.8%±4.4% were neurons (p<0.001). The rostro-caudal extent of the vector spread was approximately 3 mm (Fig. 2E). GFAP-controlled GFP expression was absent from oligodendrocytes (Fig. 2F), or neurons (Fig. 2G). In contrast, we could confirm robust GFP staining in both cortical and subcortical grey matter astrocytes (Fig. 2H). These results are illustrated in Figure 2I. The rostro-caudal spread of AAV-GFAP-GFP expression (Fig. 2J) was comparable to AAV-MBP-GFP. These results suggested that cellular promoters are sufficient to achieve widespread AAV1/2-mediated transgene expression to glia, extending over the target region into more distant areas, confirming previous findings utilizing AAV8 in adults [7].

### Transgene expression is restricted to astroglia following AAV-GFAP-GFP delivery to the neonatal brain

**Figure 3:**
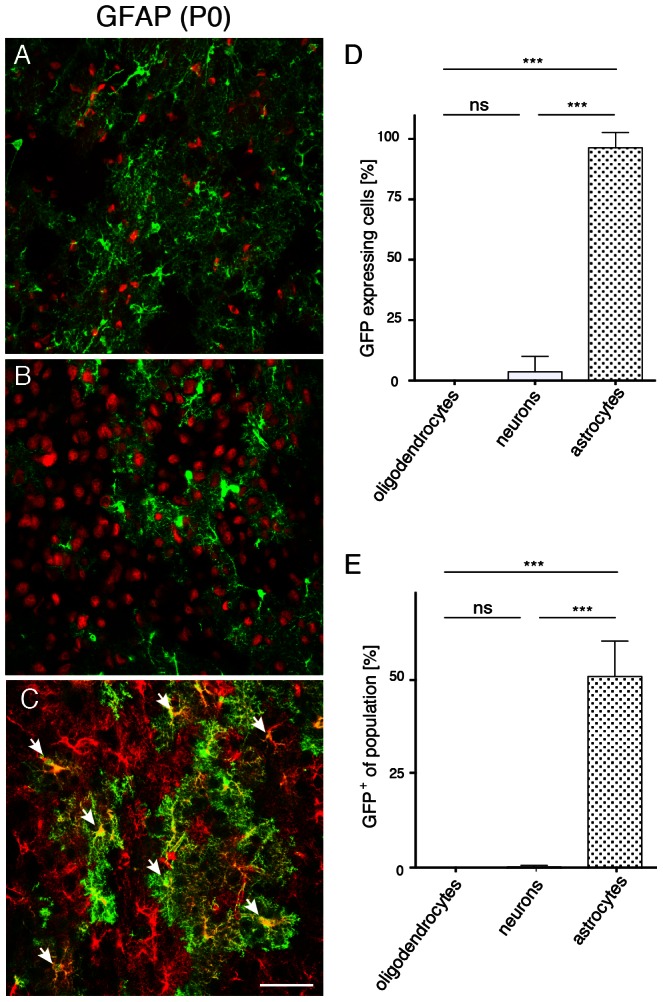
Astrocytic transgene expression after neonatal delivery of AAV-GFAP-GFP. AAV (2×10^9^ vg) was administered to the striatum of newborn mice. Brains (n = 3) were analyzed three weeks later for GFP expression (green) in combination with cell-type specific markers (red). Immunoreactivities of GFP with ASPA (A), or NeuN (B) segregated. C, Co-staining with ALDH1L1 identified GFP^+^ cells as astrocytes (arrows). D, Quantitative comparison of neural populations expressing GFP shows selectivity of the GFAP promoter in astrocytes. E, Percentage of GFP^+^ cells among individual neural populations in the target region showing transgene expression in 50% of astrocytes. In contrast, only negligible numbers of oligodendrocytes or neurons expressed the transgene. Bar: 50 µm.

**Figure 4:**
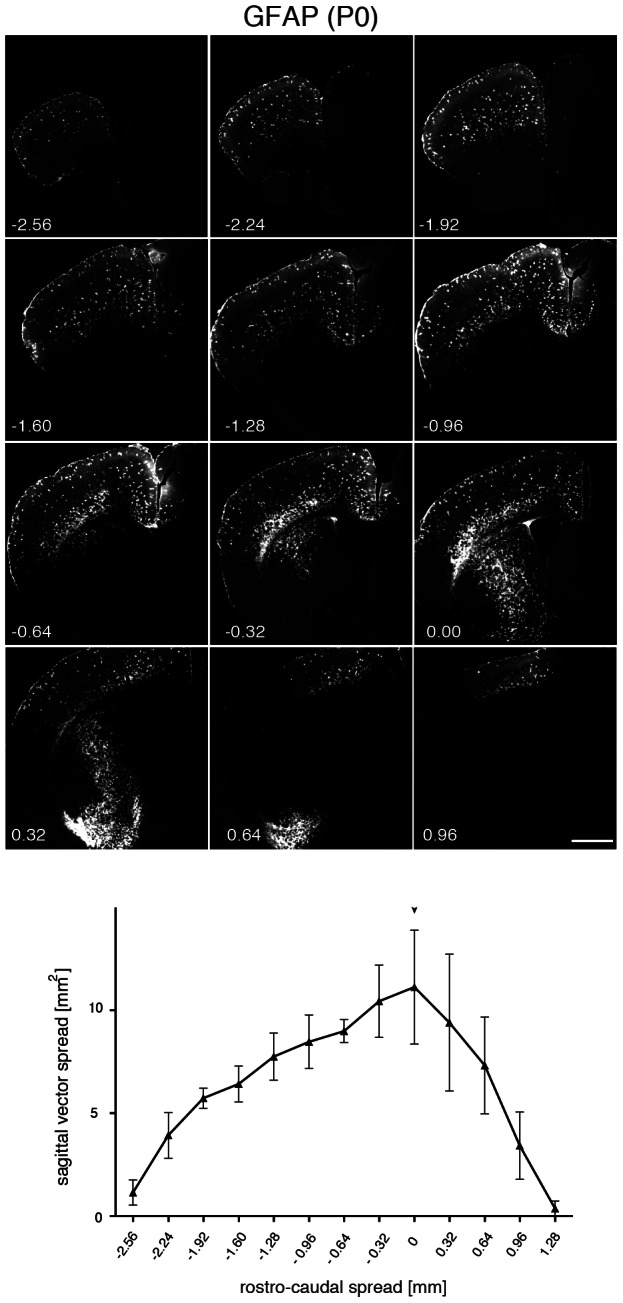
Spread of GFP expression after AAV-GFAP-GFP delivery to the neonatal striatum. The vector (2×10^9^ vg) was delivered unilaterally to the striatum (n = 3). Three weeks later brains were processed into 40 µm sections, immunostained for GFP and every 8^th^ section was used to determine the area showing GFP immunoreactivity. The graph shows quantitative results after plotting the area covered by GFP immunoreactivity as a function of the distance from the injection site. An arrowhead labels the approximate injection site. Bar: 1 mm.

We then investigated the properties of the GFAP promoter in brain sections obtained three weeks after intracranial AAV-injection to neonates (P0). GFP in the striatum was virtually absent from oligodendrocytes (Fig. 3A), or neurons (Fig. 3B), but enriched in astrocytes (Fig. 3C). Quantification of these results (Fig. 3D) showed that 96.3±3.7% of all GFP^+^ cells were astrocytes, and 3.7±3.7% were neurons (p<0.001). Transgene expression in oligodendrocytes was not observed. Figure 3E shows that 52.6±5.6% of all astrocytes but only 0.3±0.3% of neurons in the target area were GFP-positive (p<0.001). The rostro-caudal extent of the vector spread was 3.8 mm (Fig. 4).

### Preferential transgene expression in astrocytes following AAV-MBP-GFP delivery to the neonatal brain

**Figure 5:**
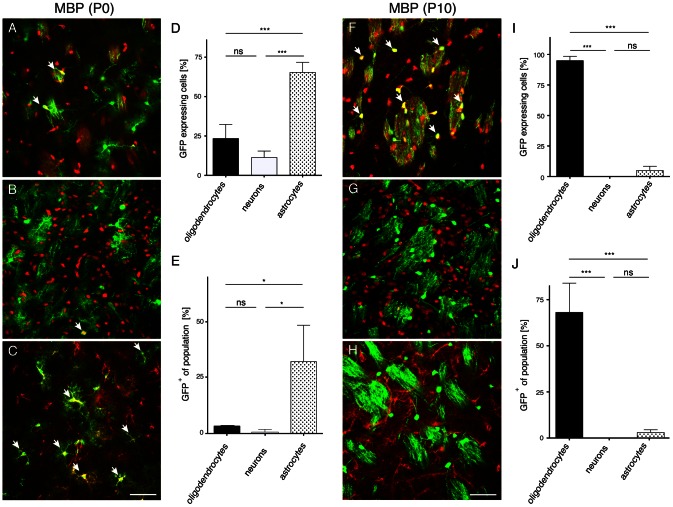
Quantification of MBP promoter selectivity after intrastriatal AAV delivery to neonates or P10 animals. AAV-MBP-GFP (2×10^9^ vg) was delivered to the striatum of neonates (A–E) or P10 animals (F–J). Brains (n = 3) were analyzed three weeks later for GFP expression (green) in combination with cell-type specific markers (red). After neonatal delivery transgene expression was observed to various degrees in oligodendrocytes (A), neurons (B), and astrocytes (C). Quantitative comparison of neural populations expressing GFP shows strongest activity of the MBP promoter in astrocytes (D). Percentage of GFP^+^ cells among individual neural populations in the target region showing transgene expression in 30% of astrocytes. Only few oligodendrocytes or neurons expressed the transgene (E). After P10 delivery transgene expression was predominantly observed in oligodendrocytes (F), but not in neurons (G), or astrocytes (H). Quantitative comparison of neural populations expressing GFP shows high specificity of the MBP promoter for oligodendrocytes (I). Percentage of GFP^+^ cells among individual neural populations in the target region showing transgene expression in the majority of oligodendrocytes. Other cell-types showed negligible percentage of GFP^+^ cells (J). Arrows in A–C and F indicate co-labelling. Bars: 50 µm.

Transgene expression driven by the MBP promoter was investigated using brain sections obtained three weeks after intracranial AAV-injection to neonates (P0). Only few GFP^+^ cells in the striatum were oligodendrocytes (Fig. 5A), or neurons (Fig. 5B). Most GFP immunoreactivity was found in astrocytes (Fig. 5C). Quantification of these results (Fig. 5D) showed that 56.3±3.7% of all GFP^+^ cells were astrocytes, compared to 11.3±2.4% neurons (p<0.001), and 23.3±5.2% oligodendrocytes (p<0.001). Figure 5E shows that 32.3±9.5% of all astrocytes, 3.0±0.2% of oligodendrocytes, and 0.8±0.5% of neurons in the target area expressed GFP (p<0.05).

### Selective transgene expression in oligodendrocytes following AAV-MBP-GFP delivery to the P10 brain

**Figure 6:**
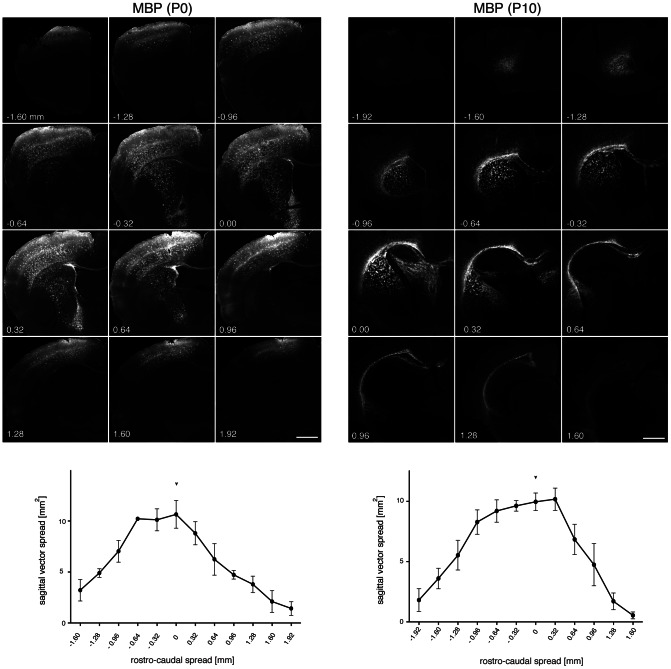
Spread of GFP expression after AAV-MBP-GFP delivery to the neonatal or P10 striatum. Vectors (2×10^9^ vg) were delivered unilaterally to the striatum (n = 3) at P0 (left) or P10 (right). Three weeks later brain sections were immunostained for GFP and every 8^th^ section was used to determine the area showing GFP immunoreactivity. The graph shows quantitative results after plotting the area covered by GFP immunoreactivity as a function of the distance from the injection site. An arrowhead labels the approximate injection site. The spread of transgene expression is comparable after neonatal or P10 delivery. Bars: 1 mm.

The poor selectivity of the MBP promoter after neonatal AAV-delivery prompted us to investigate P10 as an additional time point for AAV-MBP-GFP injection. We hypothesised that increased numbers of mature oligodendrocytes present at that stage would improve the ratio of GFP-expressing oligodendrocytes three weeks later when brains were analysed. In fact, intrastriatal AAV-MBP-GFP injection at P10 resulted in strong preponderance of GFP-expressing oligodendrocytes (Fig. 5F). In contrast, virtually no GFP immunoreactivity was detected in neurons (Fig. 5G), or astrocytes (Fig. 5H). Quantification of these results (Fig. 5I) confirmed that 96.4±1.7% of all GFP^+^ cells were oligodendrocytes, compared to 3.6±1.7% astrocytes (p<0.001). Figure 5J shows that 68.3±9.2% of all oligodendrocytes, and 2.7±0.9% of all astrocytes in the target area were GFP^+^ (p<0.001). Low power images following AAV-MBP-GFP injections at P0 or P10, showed similar spread of transgene expression (Fig. 6). However, neonatal vector delivery conferred transgene expression in forebrain grey matter, while abundant GFP immunoreactivity was observed in subcortical white matter following AAV-injection at P10.

### The developmental stage of the postnatal brain at the time of AAV injection predicts the spread of transgene expression

**Figure 7:**
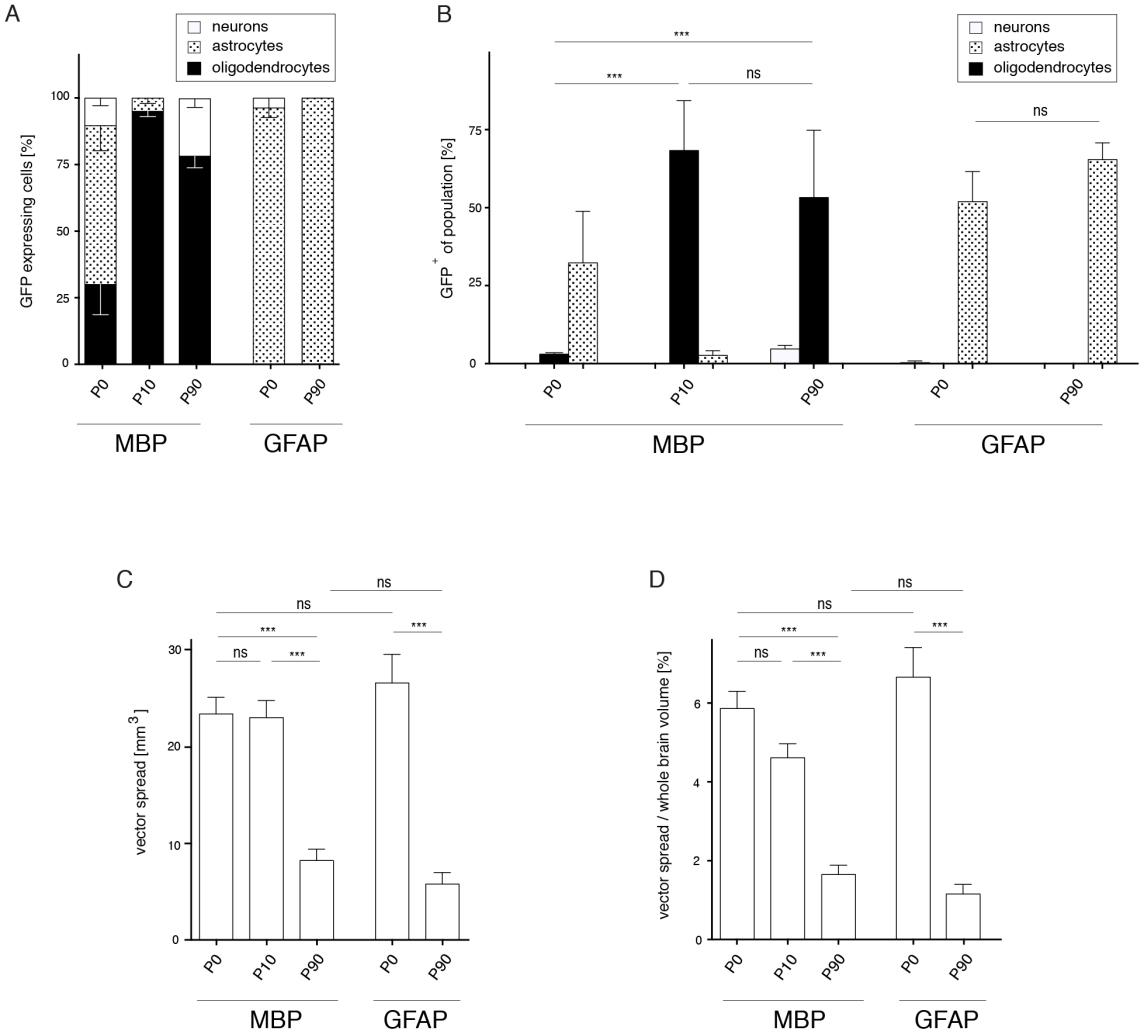
Promoter specificity and volume of GFP-expression after AAV injection at different postnatal stages. A, For each promoter and time point used, the proportion of neurons, oligodendrocytes, and astrocytes was calculated as a percentage of the total number of cells expressing GFP (n = 3). B, Summary of the percentage of GFP-expressing cells relative to the three different neural populations in the target area. Contrary to neonatal AAV-delivery, the MBP promoter robustly targeted the oligodendrocyte population following injection at P10 and P90 (MBP-P0: 3.0±0.2%, MBP-P10: 68.3±9.2%, MBP-P90: 53.3±12.5%). The GFAP promoter resulted in robust transgene expression in the astrocyte population regardless of the time point of AAV-injection (GFAP-P0: 52.0±5.6%, GFAP-P90: 65.4±3.1%). C, Volume showing GFP expression after AAV-MBP-GFP or AAV-GFAP-GFP delivery. Vector delivery at early time points result in higher efficacy compared to the adult stage (MBP-P0: 23.5±1.7 mm^3^, MBP-P10: 23.1±1.8 mm^3^, MBP-P90: 8.3±1.2 mm^3^, GFAP-P0: 26.7±5.8 mm^3^, GFAP-P90: 5.8±1.2 mm^3^). D, Vector spread relative to the whole brain volume (MBP-P0: 5.9±0.4%, MBP-P10: 4.6±0.4%, MBP-P90: 1.7±0.2%, GFAP-P0: 6.7±50.7%, GFAP-P90: 1.2±0.2%). p<0.001, 2-way ANOVA and Bonferroni post-test.

Figure 7A summarises the results for selective transgene expression by the MBP or GFAP promoter following AAV1/2-delivery at different time points during postnatal brain development. While the GFAP promoter drove specific transgene expression in astrocytes irrespective of the time point of AAV-delivery, differences for the MBP promoter selectivity were dramatic. Figure 7B summarizes the percentage of GFP-expressing cells relative to the three different neural populations in the target area. Comparison of the estimated volume of transgene expression (Fig. 7C) indicated that vector delivery to the immature brain coincides with the vector spread. No significant differences were observed between P0 and P10 irrespective of the promoter. The same was true for P90 injections. In contrast, comparison of immature (P0 or P10) with mature (P90) developmental stages at vector delivery revealed significant differences. Similar results were obtained by analysis of the vector spread relative to the whole brain volume (Fig. 7D). In conclusion, of the time points tested, P10 delivery (for oligodendrocytes) and P0 infusion (for astrocytes), was optimal for targeting transgene expression in glia.

### Selective and widespread transgene expression in oligodendrocytes following AAV-MBP-GFP delivery to the brain of a leukodystrophy model

**Figure 8:**
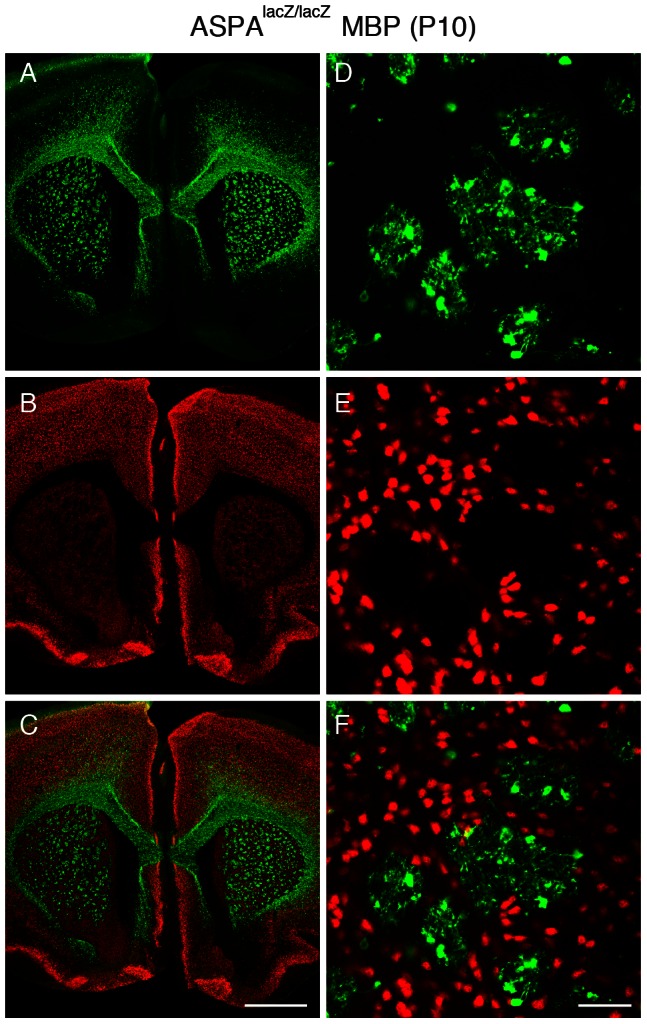
The MBP promoter is selective for oligodendrocytes in a mouse model of the leukodystrophy Canavan Disease. AAV-MBP-GFP was administered to the striatum of ASPA-deficient mice at P10 and brains analysed three weeks later to determine spread and selectivity of transgene expression. Immunodetection of GFP^+^ cells (A) and counterstaining for NeuN (B) revealed robust GFP-staining in subcortical white matter tracts. While GFP immunoreactivity (green) mostly segregated from neurons (red), the merged image revealed some co-expressing cells in the neocortex (C). High power images of the striatum showed GFP-expression exclusively in white matter oligodendrocytes (D–F). Shown are representative results of three independent experiments. Bars: C, 1 mm; F, 50 µm.

Our data indicated that AAV-mediated transgene expression can be achieved by the MBP promoter in healthy oligodendrocytes but future applications will utilise this vector platform as delivery agent for somatic gene transfer to the diseased CNS that may be less permissive for transduction by AAV. Therefore, we performed a pilot experiment involving AAV-MBP-GFP injection to the P10 brain of mouse mutants lacking aspartoacylase (ASPA). Homozygous ASPA^lacZ/lacZ^ mice [17] model Canavan Disease, an early onset leukodystrophy, characterised by oligodendroglial pathology and demyelination. Figure 8 shows representative results after double-staining to detect GFP (Fig. 8A) and neurons (Fig. 8B) indicating widespread and oligodendrocyte-specific transgene expression in the whole brain, enriched in cortical and subcortical grey matter as well as in subcortical white matter. While the merged image (Fig. 8C) shows some limited GFP immunoreactivity in cortical neurons, high power images of the striatum (Fig. 8D–F) illustrate that GFP immunoreactivity generally segregates from NeuN immunoreactivity and is restricted to oligodendrocytes. Finally, we then examined the efficacy of AAV-MBP-GFP in the human oligodendroglial cell line MO3.13 and detected robust transgene expression in these cells (Figure S2). These data indicate that this AAV system might be suited for the treatment of leukodystrophies, a group of hereditary disorders caused by oligodendrocyte dysfunction.

## Discussion

Numerous factors have been reported to influence the tropism of AAV vectors in the CNS, including the kind of recombinant genome [22], the stage of brain maturation [23], route of administration [4], vector titre [3], serotype [24], and target area [7]. However, the most significant influence for AAV-mediated cell type-specific transgene expression is conveyed by the promoter [7], [8], [25]. Surprisingly, this effective yet simple concept, so far only applied in adult rodents, has not been followed up. The aim of the current study was to accomplish targeted and widespread glial transgene expression following intracranial AAV delivery to the developing CNS. Our study design entailed packaging of GFP-expression cassettes, that only differed in the promoter, into chimeric AAV1/2. In our hands, the CBA, MBP, and GFAP promoters drove GFP expression in the same neural compartments with similar vector spread as previously reported for AAV8 [7].

To our knowledge, we provide, for the first time, experimental evidence that cellular promoters are essential to confer long-term expression in glia following AAV vector infusion to the immature brain. While the standard duration of our experiments was three weeks after AAV injection, we observed selective and widespread three-dimensional transgene expression that extended over the target region even after nine months following vector delivery (our unpublished observations). Despite small number of biological replicates, the data were clear, consistent and yielded robust statistics. Selectivity of the GFAP promoter was excellent following adult or neonatal vector delivery. It should be pointed out that grey matter astrocytes were preferentially targeted while white matter astrocytes were largely spared. The MBP promoter yielded unexpected results in that it largely drove astroglial transgene expression following neonatal AAV delivery. In contrast, this promoter was highly selective for oligodendrocytes after vector infusion at P10. This developmental dependence of the MBP promoter tropism may be influenced by age-specific differences of the vector dose/kg ratio or by the infusion parameters. Moreover, transduction of glial progenitor cells by AAV-MBP-GFP is a likely scenario given that glial differentiation is a postnatal process in the mouse. Although hypothetical, an intrinsic event in glial progenitors might irreversibly modify the recombinant MBP promoter, thereby changing its selectivity. At P10, this event might have passed, allowing for transduction of more committed oligodenroglial lineage cells and hence increased promoter selectivity in oligodendrocytes. As an example for DNA modifications impacting on transcriptional activity, silencing of AAV-mediated transgene expression by methylation of the cytomegalovirus (CMV) promoter in the brain has been described [26]. Future studies will employ a higher temporal resolution to address the potential transient transgene expression in progenitor cells.

Cell-type specific targeting using cellular promoters in the context of timed AAV gene delivery is straight forward, efficient, fast, and widely applicable. The obvious bottleneck for successful expansion of this approach is the identification of novel recombinant promoters. The size of a cellular promoter normally correlates with the specificity of transcriptional control. However, the known packaging limit of AAV constructs is <5.1 kb [27] which in turn limits promoter length and hence selectivity. We used the 2.2 kb version of the human GFAP promoter in the current study. However, shorter variants of this promoter have been described to drive transgene expression faithfully in astrocytes of transgenic mouse lines [28], and hence might represent viable adaptations for the AAV system. A number of short, yet specific neuronal promoters have already been utilised in the AAV context [16], [29]. However, there is currently no appropriately sized, oligodendrocyte-specific alternative to the 1.3 kb and 1.9 kb MBP promoter variants used in this and previous studies.

In the advent of the characterisation of novel AAV serotypes derived from non-human primates, the systemic route of administering AAV vectors for genes to the CNS has become the subject of intense study [22], [23], [29]. While the peripheral injection of AAV vectors represents a non-invasive approach, and was shown to be effective in disease models of neurological disorders [30], it elicits immunological responses and translation is hampered by issues of up-scaling vector production for use in humans [31]. Therefore, the characterisation of parameters influencing transgene expression following direct CNS injections remains a valid aspect of preclinical research.

In this regard it is important to point out that we observed improved vector spread following delivery at immature vs. mature stages, irrespective of the promoter. This could be explained by facilitated vector diffusion when the neuropil is less densely packed. Another reason might be the transduction of glial progenitor cells, enriched at earlier time points, that proliferate and lead to amplification of numbers of transgenic cells at the time of analysis. Superior specificity of transgene expression was observed following injection at P10 compared with P0 when the MBP promoter showed substantial leakiness. While promoter specificity was the focus of our study, we cannot rule out a developmental expression of an AAV receptor that permitted oligodendrocyte transduction at the later age. Our data show that the MBP promoter confers excellent anatomical spread and targeted GFP-expression in oligodendrocytes following AAV infusion at P10. This developmental stage of the mouse CNS translates to the start of the last prenatal trimester in humans [32]. Leukodystrophies, a group of childhood CNS disorders mainly caused by oligodendroglial dysfunction, may be targeted by AAV gene therapy utilizing appropriate promoters for select transgene expression. In fact, the extent of vector spread and specificity for oligodendrocytes was replicated in a leukodystrophy mouse model showing comparable results to naïve mice. Finally, adding to the potential translational relevance of our work, we detected AAV-MBP-mediated transgene expression in a model of immature human oligodendrocytes. In summary, we have optimised the timing of intervention for translating the concept of promoter selectivity utilising the AAV toolkit. This approach will be useful for the production of new disease models and genetic treatment of glial pathology, or whenever efficient gene expression in glial cells is required.

## Supporting Information

Figure S1 **MBP and CBA promoters show complementary activity in oligodendrocytes and neurons.** Enriched oligodendrocyte cultures were transfected with AAV plasmids driving GFP under the control of the CBA promoter (A–C) or the MBP promoter (D–F) followed by immunocytochemical detection of the reporter. A–C, Expression of CBA-driven EGFP is limited to L1-positive neurons. D–F, MBP-driven GFP is exclusively expressed in O4-positive oligodendrocytes. Shown are representative results of three independent experiments. Bar: 10 µm.
(TIF)

Figure S2 **AAV-MBP-EGFP mediated transgene expression in human oligodendroglial cells.** Representative image of MO3.13 cells eight days after plating and infection with (1×10^9^ vg) AAV-MBP-GFP. A, Phase contrast picture. B, Immunocytochemical detection of the reporter reveals transgene expression in cells that display an immature morphology. Bar: 150 µm.
(TIF)
