# Supplementary figures and images for: Glial Promoter Selectivity following AAV-Delivery to the Immature Brain
Source: PLoS One. 2013 Jun 14;8(6):e65646. doi: 10.1371/journal.pone.0065646 (PMC3683058; doi:10.1371/journal.pone.0065646)

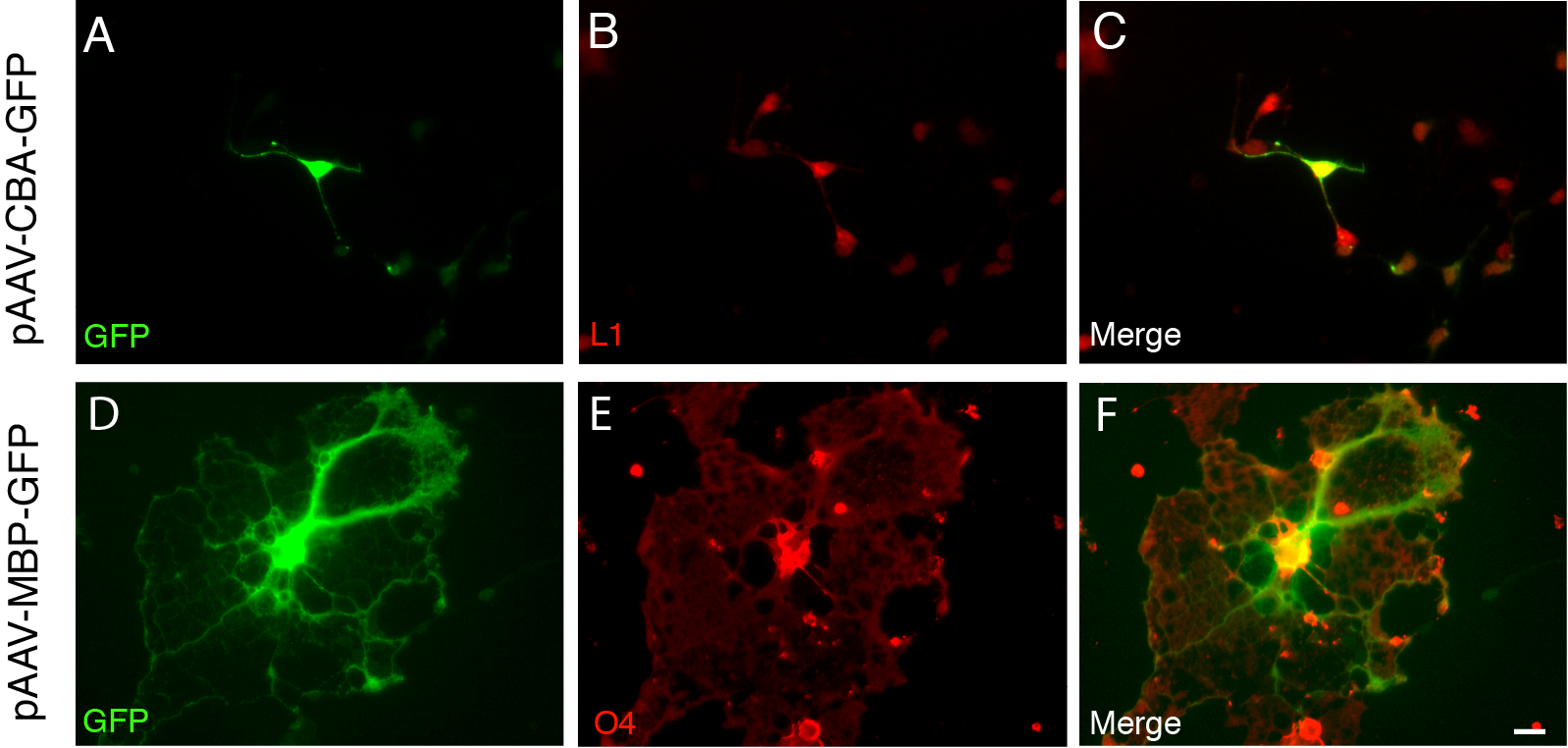

Supplement: Figure S1 — MBP and CBA promoters show complementary activity in oligodendrocytes and neurons. Enriched oligodendrocyte cultures were transfected with AAV plasmids driving GFP under the control of the CBA promoter (A–C) or the MBP promoter (D–F) followed by immunocytochemical detection of the reporter. A–C, Expression of CBA-driven EGFP is limited to L1-positive neurons. D–F, MBP-driven GFP is exclusively expressed in O4-positive oligodendrocytes. Shown are representative results of three independent experiments. Bar: 10 µm. (TIF) [file pone.0065646.s001.tif]

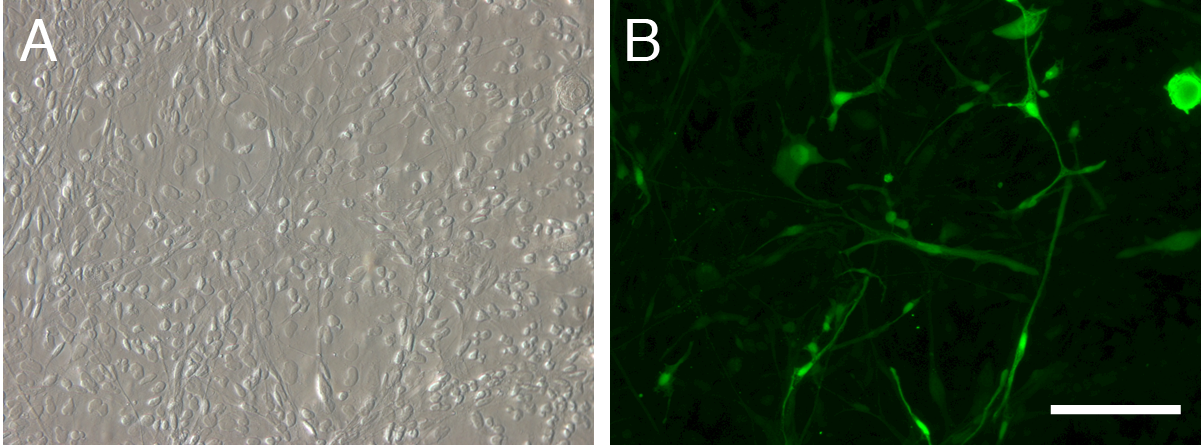

Supplement: Figure S2 — AAV-MBP-EGFP mediated transgene expression in human oligodendroglial cells. Representative image of MO3.13 cells eight days after plating and infection with (1×10^9^ vg) AAV-MBP-GFP. A, Phase contrast picture. B, Immunocytochemical detection of the reporter reveals transgene expression in cells that display an immature morphology. Bar: 150 µm. (TIF) [file pone.0065646.s002.tif]
